# Comparison of gait in progressive supranuclear palsy, Parkinson’s disease and healthy older adults

**DOI:** 10.1186/1471-2377-12-116

**Published:** 2012-10-02

**Authors:** Thorlene Egerton, David R Williams, Robert Iansek

**Affiliations:** 1 National Parkinson Foundation Center of Excellence, Clinical Research Centre for Movement Disorders and Gait and Victorian Comprehensive Parkinson’s Program, Kingston Centre, Cheltenham, VIC, Australia; 2 Van Cleef Roet Centre for Nervous Diseases, Monash University, Clayton, VIC, Australia; 3 Clinical Research Centre for Movement Disorders and Gait, Kingston Centre, Warrigal Road, Cheltenham, VIC, 3192, Australia

**Keywords:** Progressive supranuclear palsy, Parkinson’s disease, Gait, Older people

## Abstract

**Background:**

Progressive supranuclear palsy and Parkinson’s disease have characteristic clinical and neuropathologic profiles, but also share overlapping clinical features. This study aimed to analyze the gait of people with progressive supranuclear palsy (n=19) and compare it with people with Parkinson’s disease (n=20) and healthy older adults (n=20).

**Methods:**

Gait was recorded at self-selected preferred, fast, very fast, slow and very slow speeds. Stride length was normalized to leg length. Linear regression analyses were carried out between cadence and stride length. Other gait variables were compared for each participant’s ‘walk’ which had stride length closest to 1.4.

**Results:**

All groups showed a strong linear relationship between stride length and cadence with no difference between groups (p>0.05). The intercept between cadence and stride length was lowest in the progressive supranuclear palsy group and highest for older adults (p<0.001). The progressive supranuclear palsy group had higher cadence than older adults (p>0.05), and greater step width and greater double support phase compared with the other two groups (p<0.05).

**Conclusions:**

The temporal-spatial gait characteristics of progressive supranuclear palsy and Parkinson’s disease are largely similar, with similar disruption to scaling of stride length. The additional findings of increased step width and double support percentage suggest increased severity of gait abnormality compared to Parkinson’s disease, despite similar disease duration. The findings are consistent with the clinical features of greater instability and more rapid disease progression in progressive supranuclear palsy compared to Parkinson’s disease and implicates the early pathological involvement of brain regions involved in gait control.

## Background

Progressive supranuclear palsy (PSP) [1] is the second most common cause of neurodegenerative parkinsonism, and causes progressive deterioration in motor and subcortical cognitive function. The neuropathologic features of PSP include marked midbrain atrophy and atrophy of the pallidum, thalamus, subthalamic nucleus and frontal lobes [2]. Clinically, PSP is a degenerative movement disorder with a variable clinical presentation that includes vertical gaze palsy, pseudobulbar palsy, axial rigidity, and cognitive impairment. Patients typically have bradykinesia, the appearance of a fixed stare and a soft, slurred, growly voice [3]. Gait disturbance is recognized as one of the core diagnostic criteria and may present as short, shuffling steps, gait freezing, lurching unsteady gait or spontaneous falls, which may become the dominant features [3,4]. Diagnosis can be difficult and often takes several years due to the variability of presenting signs and symptoms and frequency of atypical presentations [3].

In the early stages, PSP can be difficult to distinguish from PD as both patients develop hypomimia, shuffling gait, freezing and bradykinesia [5]. The forward flexed posture of PD is often not present in PSP, and falls in PSP tend to be backwards [6]. Although gait changes are an important clinical feature of PSP, it has not been systematically analyzed and compared to patients with PD. Assessing the temporal-spatial characteristics of gait, and comparing to people with PD and healthy older adults (HOA), may help to identify distinguishing features of PSP, permit further understanding of gait control pathways affected by PSP and in the longer term contribute to the development of more effective, disease-specific treatment options.

We sought to evaluate the temporal-spatial gait characteristics in patients with PSP, and to compare with the gait of people with hypokinetic gait due to PD and healthy older adults. In this cross sectional study we aimed to characterize gait using the stride length versus cadence relationship (SLCrel) and parameters which relate to dynamic postural instability [7-10] and hypokinesia [11,12].

## Methods

Nineteen people with clinically diagnosed probable PSP [13] and 20 people with clinically diagnosed PD were recruited from Movement Disorder Specialty Clinics. PD patients were excluded if they experienced at least moderate dyskinesias. Participants were tested on their usual medications. The 20 HOA were recruited by convenience from our laboratory database and the local community. All participants were required to be able to walk at least 10m without physical assistance or use of a walking aid, and achieved a Mini Mental State Examination score of greater than 23/30 [14]. Potential participants were excluded if they suffered from any other disease to a degree that would likely effect their gait (for example, lower limb or back pain during walking, hemiplegia from stroke, peripheral neuropathy secondary to diabetes). All participants gave their written informed consent and approval for the study was gained from the Southern Health Research Ethics Committee.

The footstep patterns of participants were recorded using a GAITRite® walkway system (CIR Systems Inc., Havertown, PA) at self-selected speeds of very slow, slow, preferred, fast and very fast. Gait variables determined from GAITRite® have previously been shown to be valid and highly reliable [15,16]. Three trials of each speed were recorded with only the second and third being used in the analysis. Trials at ‘preferred’ speed were always recorded first to prevent subtle influence from faster or slower walks. The remaining four speed levels were performed in the order slow, very slow, fast and finally very fast, or, fast, very fast, slow and finally very slow. Participants were randomly allocated one of the two orders. Close supervision was provided by experienced clinicians, and a safety belt was worn by most PD and PSP participants.

Data screening and sometimes cropping of walks was performed to ensure there were no data errors or anomalies in the walks such as stopping on the mat, festinating, or stepping off the side of the mat. Each walk used in the analysis had a minimum of eight consecutive steps. Speed normalized to leg length (nSp), stride length normalized to leg length (nSL) and cadence, all recorded at preferred speed, were compared. For comparisons of preferred speed gait characteristics, the second and third walks at self-selected preferred speed for each participant were averaged to produce one value per participant before analysis of group data were carried out. Disease severity was measured using PSP Rating Scale for PSP participants (0 = no symptoms – 100) and Unified Parkinson’s disease Rating Scale – motor subsection (UPDRS III) for PD participants (0 = no symptoms – 56). Other gait variables of interest were speed dependent, therefore the walk that resulted in nSL closest to 1.4 (the mean nSL at preferred speed for the cohort, SD = 0.26) was identified and used for further gait comparisons. Similar stride length was used rather than similar speed as stride length is a key determinant of speed, which in turn is the combination of two variables. The variables compared at comparable stride lengths were cadence, double support percentage of gait cycle (DS%), step width, stance time variability, and stride length variability. Variability measures were calculated as the standard deviation across all steps of the selected walk.

The minimum, maximum and range of both stride length and cadence were determined for each individual from the second and third walks at each of the five speed conditions (ten walks). The nSL versus cadence relationship (SLCrel) was examined using methods developed by our group and previously described [12]. The slope, intercept and R^2^ values were calculated from linear regression using SPSS v18 (Chicago, IL). For this analysis, walks with cadences of greater than 150 steps/min were removed as we have previously found that after removing walks with these very high cadences, almost all (95%) SLCrels for healthy adults were strongly linear (*R*^2^ ≥ 0.90) rather than quadratic [12]. The intercept was calculated at a cadence of 100steps/min to ensure it was within the data range and more accurately reflected the gain of the SLCrel than using an intercept at a cadence of zero [12,17]. All comparisons between the groups for each variable were carried out using one-way ANOVA tests with Bonferroni post hoc analyses (SPSS v18, Chicago, IL).

A regression line representing the SLCrel for each group as a whole was also constructed using all the individual data points from walks with cadences < 150 steps/min. The group slopes and intercepts (again at a cadence of 100steps/min) from these plots are also reported.

## Results

Demographic information is shown in Table 1. The HOA participants were more likely to be female and weighed less but there were no differences between the groups in age or height. There was no significant difference between the PSP and PD groups in mean symptom duration, PSP 4.3 years (SD = 2.7) versus PD 6.6 years (SD = 5.8), or mean time since last levodopa medication for those on medication, PSP (n = 9, 50% of sample) 3.0 h (SD = 1.8) versus PD 2.2 h (SD = 1.5). The mean PSP Rating Scale score for the PSP participants was 41.7/100 (SD = 17.2), while the mean UPDRS III score for the PD participants was 14.4/56 (SD = 16.7).

**Table 1:** Demographic information

	**PSP*(n = 19)***	**PD*(n = 20)***	**HOA*(n = 20)***	
**Age, yrs**	69.1 (8.7)	68.3 (7.9)	71.8 (4.1)	*n/s*
**Male, n(%)**	16 (84)	16 (80)	7 (35)	*P* = 0.001
**Height, cm**	170.6 (8.2)	171.8 (8.0)	166.6 (6.3)	*n/s*
**Weight, kg**	80.5 (13.5)	78.3 (10.2)	68.4 (11.5)	*F*_2_,_56_ = 5.9, *P = 0.01*
**Symptom duration, yrs**	4.3 (2.7)	6.6 (5.8)	-	*n/s*
**Disease severity**	41.7 (17.2)	14.4 (16.7)	-	-
**Normalized speed**	1.03 (0.27)	1.28 (0.22)	1.54 (0.16)	*F*_2_,_56_ = 25.8, *P < 0.001*
**Normalized stride length**	1.2 (0.2)	1.4 (0.2)	1.6 (0.1)	*F*_2_,_56_ = 25.6, *P < 0.001*
**Cadence (steps/min)**	103.6 (9.3)	109.7 (6.9)	113.4 (7.0)	*F*_2_,_56_ = 7.9, *P = 0.001*
Cadence (steps/min)*	119.8 (15.4)	108.9 (18.9)	92.7 (9.5)	*F*_*2,56*_*= 15.9, P <0.001*
DS (%)*	31.1 (4.6)	27.9 (3.0)	25.6 (2.7)	*F*_2,56_ = 12.2, *P < 0.001*
Step width (cm)*	11.4 (4.2)	9.0 (2.9)	8.5 (2.9)	*F*_2,56_ = 4.0, *P = 0.02*
Stance time variability (sec)*	0.024 (0.009)	0.018 (0.008)	0.025 (0.013)	*n/s*
Stride length variability (cm)*	3.2 (1.7)	2.4 (1.2)	2.8 (1.6)	*n/s*

Mean (standard deviation, SD) for each group. Disease severity was measured using PSP Rating Scale for PSP participants (0=no symptoms – 100) and Unified Parkinson’s Disease Rating Scale – motor subsection (UPDRS III) for PD participants (0=no symptoms – 56). Mean (SD) normalized speed, normalized stride length and cadence for preferred speed walks. Mean (SD) of other gait parameters for the walk with normalized stride length closest to 1.4.

* Determined from walk with normalized stride length closest to 1.4. *PSP* progressive supranuclear palsy, *PD* Parkinson’s disease, *HOA* healthy older adult, *DS* double support percentage of gait cycle, *n/s* not statistically significant.

**Figure 1:**
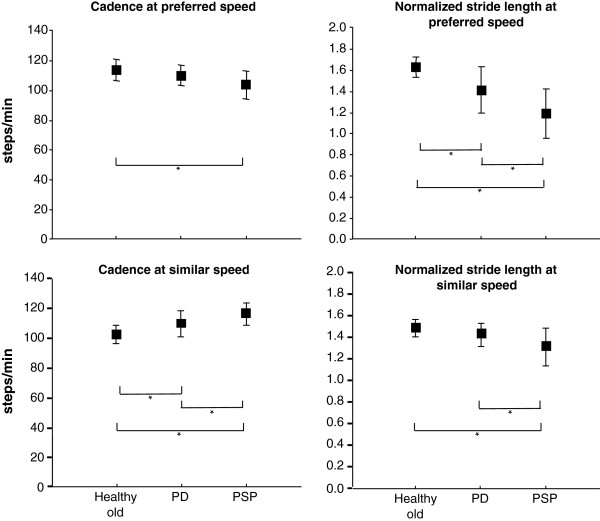
**Comparison of gait between groups.** Mean ± 1SD for each group for cadence and normalized stride length at preferred speed, and double support % of gait cycle and step width at similar stride lengths. *denotes significant difference between groups.

At self-selected preferred speed, the PSP participants walked slower and with shorter stride length than both PD and HOA participants (Table 1). They had lower cadence than HOA only. Figure 1 illustrates that the different mean stride lengths account for more of the differences in speed than cadence.

Walking with similar stride length, the PSP and PD participants had significantly higher cadence than the HOA group (Table 1). Significant group differences were also found for DS% and step width, where the PSP group differed from both PD and HOA groups, PSP having significantly greater DS% and wider step width (Figure 1 and Table 1). There were no significant group differences for stride length variability or stance time variability.

**Figure 2:**
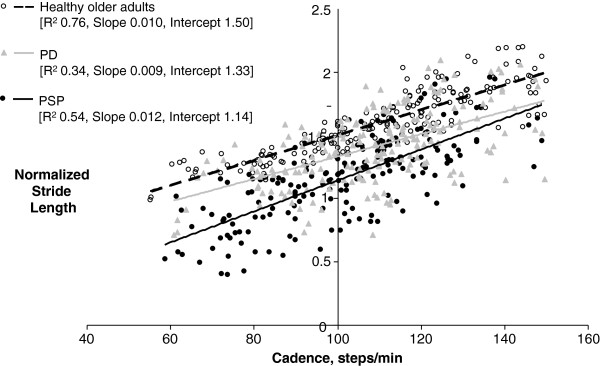
**Stride length versus cadence.** The normalized stride length versus cadence data points and regression lines for all the walks up to cadence = 150steps/min for all participants in each group.

The SLCrel group data are presented in Table 2. All SLCrels were examined and none were found to be other than linear. Two PDs had very limited range of SL which resulted in low R2 values (0.23 and 0.22). All other participants had R2 values of > 0.83. The slope and intercept values of these two outlying PDs were excluded from the analysis. Differences between the groups were found for SLCrel intercept which was lowest for PSP and highest for HOA, minimum and maximum stride lengths, which were both lowest for PSP and highest for HOA, and maximum and range of cadence, which were both only different between PSP and HOA, with PSP having lower maximum cadence and smaller cadence range than HOA. The SLCrel plots for all data points in each group are shown in Figure 2.

**Table 2:** Summary gait data

	**SLCrel slope**	**SLCrel intercept**	**SLCrel*R***^**2**^	**Min nSL**	**Max nSL**	**Range nSL**	**Min cad**	**Max cad**	**Range cad**
**PSP**	0.011 (0.004)	1.14 (0.20)	0.92 (0.08)	0.9 (0.3)	1.4 (0.3)	0.57 (0.21)	74.9 (9.7)	128.9 (13.5)	54.1 (18.2)
**PD***(n=18)*	0.011 (0.004)	1.35 (0.22)	0.94 (0.06)	1.1 (0.2)	1.7 (0.2)	0.55 (0.22)	82.7 (11.6)	137.8 (12.3)	55.1 (15.1)
**HOA**	0.011 (0.005)	1.48 (0.07)	0.95 (0.04)	1.3 (0.1)	1.9 (0.2)	0.68 (0.17)	78.1 (14.0)	147.8 (12.7)	69.6 (22.1)
	*P* = 0.75	*F*_2,56_ = 17.7, *P* < 0.001	*P* = 0.15	*F*_2,56_ = 15.1, *P* < 0.001	*F*_2,56_ = 23.4, *P* < 0.001	*P* = 0.23	*P* = 0.13	*F*_2,56_ = 10.5, *P* < 0.001	*F*_2,56_ = 4.3, *P* = 0.02

Mean (SD) for each group for the measures taken from ten walks (two each at self-selected very slow, slow, preferred, fast and very fast speeds). Note the stride length – cadence relationship (SLCrel) measures excluded walks with cadence >150steps/min and two outlying PD participants’ data was excluded

*SLCrel* stride length versus cadence relationship, *nSL* normalized stride length, *cad*=cadence, *PSP* progressive supranuclear palsy, *PD* Parkinson’s disease, *HOA* healthy older adult.

## Discussion

This study provides temporal-spatial gait data for people with PSP and compares their gait with PD and HOA. Preferred speed walks showed decreased nSL to be the main contributor to slowed walking speed in both PD and PSP. PSP also had lower cadence than ‘normal’ at preferred speed. People with PSP had reduced nSL for their cadences across all the self-selected speeds (reduced intercept) indicating an alteration in the gain of the normal stride length - cadence relationship.

Comparison of ‘walks’ at similar stride length showed that people with PSP have significantly higher cadences for the stride length than HOA and more ‘abnormal’ DS% and step width than PD. The significantly increased DS% and step width may be manifestations of unsteadiness or compensations to enhance postural stability [8]. Falls are a cardinal feature of PSP and the occurrence of falls has been linked to the presence of gaze problems, axial rigidity, cognitive decline [4]. Increased DS% and step width may be an indication that dynamic instability is also a contributing factor to fall frequency in PSP. However, as DS% was also significantly greater for the PD group when compared with HOA, the finding may be a result of greater overall severity of gait abnormality, despite no difference in disease duration, among the PSP group than for PD, rather than an additional gait abnormality. This is consistent with the clinical finding of more rapid disease progression for people with PSP.

There are features of PSP that are likely to contribute to gait abnormalities. Motor causes include weakness and spasticity, which along with bradykinesia is thought to explain the ‘lurching’ style gait [3]. Axial (more so than appendicular) rigidity and dystonia are often present. The visual problems including vertical movement and convergence difficulties, reduced blinking and involuntary eyelid closing, may also lead to gait alterations as well as directly contributing to fall frequency [18][3]. Finally, many of the cognitive problems that are features of PSP may contribute to gait instability, including apathy and decline of executive function, reduced processing speed, impaired attention, and diminished working memory.

Callisaya et al. [19] found that the speed of the walk explained most of the variation in the temporal variability found among older people. This, they presume, is because walking at slower speeds disrupts the temporal automaticity of gait. In this study, we controlled for speed by selecting a self-paced walk at a target stride length. The selected walk was a slow or very slow walk for all the HOA, but was a fast or very fast walk for most (84%) of the PSP. All participants may have been influenced by the effect of non-preferred speed on variability, however stance time variability for all groups in this study were comparable to that reported for older adults, mean 0.02s (SD 0.01) [19].

This study showed that while the stride length versus cadence relationship was intact in our PSP participants, it had a lower intercept, that is, a smaller stride length was associated with a higher cadence. This was also characteristic of the PD group in this and previous studies [11], although the lower intercept in our PSP group was more severe. This suggests that people with PSP share the defective scaling of stride length that underlies gait disturbance in PD, but to a more severe extent. These findings are consistent with the concept of a mismatch in stride length selection associated with basal ganglia malfunction in both disease states. Given the upper brain stem involvement in PSP we might have expected some additional disturbance of cadence control [20]. While the PSP group had lower cadence at preferred speed and higher cadence when walks with similar stride lengths were compared, no disruption to the linkage between stride length and cadence manifest in the SLCrel. This may be expected to break down with progressive involvement of the lower brain stem, and may be present in the PSP patients who did not satisfy inclusion criteria for the present study.

The findings from this study indicate that some treatment modalities that are successful in improving function for people with PD, such as the use of cueing when walking to maintain stride length, may also be appropriate for people with PSP. However, the instability-related gait abnormalities found in this PSP group support the inclusion of additional treatment techniques to optimize their gait and maintain independent functioning for as long as possible.

The study findings need to be considered in the context of several methodological limitations. There were differences in the groups which may have an impact on the data and the conclusions that can be drawn. While disease duration was similar, it was not possible to directly compare disease severity between the two patient groups as two different scales were used. Differences in disease severity may explain some of the differences in gait between the patient groups.

The difficulty in recruiting participants with PSP who meet the inclusion/exclusion criteria for this study has resulted in low participant numbers. The testing was carried out without withholding of levodopa medication. All PD participants and half of the PSP participants were prescribed levodopa medication. Levodopa medication is known to normalize stride length and the stride length versus cadence relationship intercept for people with PD [11]. It is not known whether it achieves a similar effect on gait in PSP and some of the gait abnormalities may be underrated particularly among the PD group. While this may be seen as a limitation, the data reflects the presentation of gait abnormalities seen in clinical settings. Given that gait within patients with advanced PD fluctuates extensively and often does not follow a predictable pattern related to medication timing [21], and the clinical presentation among our small cohort of PSP patients was considerably heterogeneous, we were not able to carry out a comparison of gait between the PSP patients prescribed versus not prescribed levodopa medication. However, we recommend such a study for future research.

That there were more females in the HOA group may have impacted on the results. Gender differences are most apparent for the variables of speed and stride length where females have lower speed and shorter stride length than males [22]. That we never-the-less found significant differences between the groups in these two variables indicates that the group gender differences are unlikely to have impacted on our conclusions. Weight was previously not found to be associated with similar gait variables in a population based study of older people [19].

## Conclusions

These findings indicate that PSP pathology affects gait control pathways that are also affected in PD. Gait changes associated with defective scaling of stride length and postural instability were more marked in PSP than those with PD despite similar disease duration between the groups. This may be a manifestation of more rapid progression of the disease or damage to additional pathways in PSP.

## Competing interests

The authors declare that they have no competing interests.

## Authors’ contributions

TE contributed to the design, organization and execution of the project, carried out the statistical analysis and interpretation of the findings, and wrote the manuscript. DRW contributed to the participant recruitment, interpretation of the findings and reviewing the manuscript. RI contributed to the conception of the study, participant recruitment, design of the methods including data and statistical analysis, interpretation of the findings and reviewing the manuscript. All authors have read and approved the final manuscript.

## Pre-publication history

The pre-publication history for this paper can be accessed here:

http://www.biomedcentral.com/1471-2377/12/116/prepub
